# Self-Reported ADHD Symptoms and Interhemispheric Interaction in Adults: A Dimensional Approach

**DOI:** 10.1155/2015/254868

**Published:** 2015-05-19

**Authors:** Saleh M. H. Mohamed, Norbert A. Börger, Reint H. Geuze, Jaap J. van der Meere

**Affiliations:** ^1^Department of Clinical & Developmental Neuropsychology, University of Groningen, Grote Kruisstraat 2/1, 9712 TS Groningen, Netherlands; ^2^Department of Psychology, Beni-Suef University, Salah Salem Street, Beni-Suef 62511, Egypt

## Abstract

The present study applied the dimensional approach to test whether self-reported symptoms of Attention Deficit/Hyperactivity Disorder (ADHD) in adults are associated with the speed of interhemispheric interaction. A sample of first grade students (*N* = 112) completed Conners' Adult ADHD Rating Scales and letter matching reaction time tasks. In the tasks, participants had to match a single target letter displayed below the fixation cross, either on left or right visual field, with one of two letters displayed above the fixation cross, one letter on each visual field. For each task, identical letters were presented either within the same visual field (within hemisphere condition) or across visual fields (across hemisphere condition). Interhemispheric interaction was indexed as the difference in mean reaction time between within and across hemisphere conditions. Comorbid problems such as depression, anxiety, and stress may affect task performance and are controlled for in this study. Findings indicated that self-reported ADHD symptomology, especially hyperactivity, in the presence of stress was weakly but significantly associated with fast interhemispheric interaction.

## 1. Introduction

A recent meta-analytic review indicates that childhood and adulthood Attention Deficit/Hyperactivity disorder (ADHD: DSM-IV) are characterized by compromised information processing skills including executive function demonstrated in reaction time studies [1]. These cognitive skills are traditionally associated with prefrontal lobe functioning, and indeed, brain mapping studies reported reduced activation in fronto-parietal-cerebral areas (see, e.g., the meta-analysis of Hart and colleagues [2]). Moreover, evidence is growing that cognitive skills including executive function also rely on the cooperation between the two cerebral hemispheres subserved by the corpus callosum [3–5]. This suggests that compromised information processing skills in ADHD may be associated with inefficient interhemispheric communication.

Recent meta-analytic reviews on the structure of the corpus callosum in patients with ADHD concluded that there is some evidence that its size is reduced; however, some potential confounders have to be taken into consideration such as a small sample size; some patients were on medication during the assessment. Moreover, most of the studies included patients with comorbid disorders such as oppositional defiant disorder and conduct disorder [6, 7]. With regard to corpus callosum functioning, studies using visual stimuli reported either faster [8–10] or similar interhemispheric interaction between patients with ADHD and the norm [11, 12]. In addition, two dichotic listening studies provided some indication that interhemispheric interaction is compromised in patients with ADHD with deficits located in one hemisphere and/or poor interhemispheric interaction [13, 14].

Inconsistencies in findings might at least partly be caused by complex interactions between group factors such as age, IQ, gender, different types of comorbidities [15], handedness [16], different task characteristics, and a variety of instruments used for classification. Moreover, what the studies have in common is that they were carried out along the lines of the categorical approach; that is, the participating individuals with ADHD fulfilled the DSM criteria.

There are several reasons to prefer a dimensional approach above the categorical approach when studying the relation between speed of interhemispheric interaction and ADHD symptomatology. The dimensional approach does not require the arbitrary dichotomization of individuals into categories based on an all-or-none principle but positions individuals on a continuum [17]. Consequently, it is by definition focused on a nonclinical or a mixed population and is therefore less vulnerable to comorbidities involved in clinical ADHD and its variability in medical history [15, 18]. In addition, the approach offers a more powerful statistical test of any hypothesis because dichotomizing continuous variables results in the loss of potential useful information [15]. Consequently, the dimensional approach leads to a more accurate assessment of ADHD symptoms and provides better understanding of its etiology [19, 20].

The dimensionality of ADHD in adults has been studied on the basis of self-reports such as Conners' Adult ADHD Rating Scales (CAARS) [21], a popular instrument containing the key domains (inattention, hyperactivity, and impulsivity) and an “overall” index of ADHD symptoms. ADHD self-reports such as the CAARS are considered to be reliable and valid to estimate the symptomatology of the disorder [22–25], and scores covary with keys of ADHD such as boredom and sustained attention [26] and with comorbid problems such as depression, anxiety, and stress [27, 28]. Electrophysiological and MRI studies showed that ADHD self-reports, especially the dimension of inattention, are associated with compromised response monitoring [29] and reduced total brain volume [30]. These findings in particular underscore the neurobiological dimensionality of self-reported ADHD symptoms.

The present study is the first to investigate the relation between self-reported ADHD symptomatology and interhemispheric interaction in an adult population. ADHD symptomatology was measured by the CAARS, because it provides scores on continuum dimensions rather than other ADHD questionnaires that classify individuals into two categories (having or having not ADHD). Interhemispheric interaction was measured using the Banich tasks [31–33]. There are various divided visual field paradigms developed to measure interhemispheric interaction such as the Poffenberger, redundant gain, Banich, and Dimond paradigms [34, 35]. We chose in favor of the Banich paradigm because, compared to other visual paradigms, the Banich paradigm is most pronounced in tapping attentional demands which makes the task most sensitive for ADHD difficulties. The Banich paradigm has neuroanatomical support [36] and has been used frequently in studies on normal and various patient samples [37–41].

The Banich paradigm consists of two conditions: a within hemisphere condition (the matching letters are presented within the same visual field, and the processing is controlled by one hemisphere), and an across hemisphere condition (the matching letters are presented across visual fields; as a result, the information must cross the corpus callosum). The paradigm is based on the fact that the utility of interhemispheric interaction varies with task complexity. Increasing task complexity guides the reaction time performance to benefit from the cooperation between the two hemispheres with a faster reaction time on an across hemisphere condition compared to a within hemisphere condition. Therefore, the paradigm has two tasks that differ in complexity level: a physical-identity task and a more complex name-identity task. In the physical-identity task only perceptual identification is required to reach a decision. Here, faster or equal reaction times are expected in the within hemisphere condition compared to the across hemisphere condition, whereas, in the name-identity task, additional computational steps beyond the perceptual identification (i.e., naming) are required to reach a decision. In this case, the expectation is faster reaction times on across hemisphere condition compared to the within hemisphere condition indicating that faster interhemispheric interaction is required for complex information processing [31, 41–44].

According to the Banich paradigm, there are two ways to measure interhemispheric interaction; the first is to calculate the difference in mean reaction time between within and across hemisphere task conditions. The second is to investigate reaction times for only the across hemisphere condition. The latter option is considered to be less accurate because processing delay is not controlling for left or right hemisphere deficits [32, 33]. Therefore, the present study used the first option to estimate interhemispheric interaction.

The main research question is whether the key domains of the ADHD symptomatology included in the CAARS predict the speed of interhemispheric interaction. The study takes anxiety, depression, and stress into account since it is well-recognized that emotional state and mood symptoms are related to ADHD symptomatology, especially stress [45, 46] that causes pervasive and persistent impairments across several domains of life in subjects with ADHD [47]. Experiencing stress has been shown to negatively affect the neuronal connections in the prefrontal cortex causing poor working memory, impaired impulse control [48], and may affect the interaction between the two hemispheres [49]. All in all, reasons enough to explore the relationship between mood symptomatology (anxiety, depression, and especially stress) and interhemispheric interaction [50–53]. To this end, the participants completed the Depression, Anxiety and Stress Scales (DASS) [54].

## 2. Method

### 2.1. Participants

One hundred thirty-four undergraduate psychology students reacted to an advertisement to join a laboratory experiment about interhemispheric interaction and ADHD symptoms. With an emphasis on ADHD symptoms in the advertisement we aimed to attract a larger than usual proportion of subjects with ADHD that would result in a sample with a wide range of ADHD symptoms from zero to full diagnosis. Based on the questionnaire information the following exclusion criteria were applied: (a) reported former diagnosis with ADHD and/or depression and/or anxiety but currently reporting few ADHD symptoms (i.e., CAARS score ≤65), (b) being under medication related to ADHD and/or mood disorders, and (c) self-reported left handedness.

One hundred twelve subjects remained in the sample including 11 subjects with high scores on the ADHD index scale (i.e., ≥65). Participants reported normal or corrected to normal vision. Using Edinburgh Handedness Inventory [55], it appeared that seven participants were mixed/inconsistent-right handed.  Table 1 shows the description of the study sample.

The responsible ethical committee “Ethical Committee Psychology-University of Groningen” has approved the experiment with a research code “13008-NE.” The experiment was conducted with the understanding and the consent of the human subject. Written informed consent was obtained from all participants. Participants were informed that their responses will be kept strictly confidential and anonymous, and they have the option to withdraw from the study at any time, without penalty.

### 2.2. Questionnaires

The participants anonymously completed Conners' Adult ADHD Rating Scales (CAARS) [21] and the Depression, Anxiety and Stress Scales (DASS) [54]. The CAARS consists of 66 four-point items ranging from 0 (not all all) to 3 (very frequently), for example, “I'm absent minded in daily life activity.” The items are surveying four dimensions. Three dimensions correspond to core features of ADHD (inattention/memory problems, impulsivity/emotional lability, and hyperactivity/restlessness). The fourth dimension corresponds to an important consequence of ADHD, that is, problems with self-concept. The scale also contains the DSM-IV ADHD subscales and the ADHD index subscale. The latter measures the overall level of ADHD related symptoms. The ADHD index subscale score is seen to be the most reliable and valid estimate of self-reported overall ADHD symptomatology [27, 56, 57]. From the CAARS, an inconsistency index may be calculated that indicates inconsistent responding based on eight pairs of items has similar content; the score is computed by summing the difference scores on each pair.

The DASS questionnaire is subdivided into three subscales: (a) depression; (b) anxiety; and (c) stress. Each subscale contains 14 items (e.g., item for depression subscale: “I felt sad and depressed”; for anxiety subscale: “I felt terrified”; for stress subscale: “I found it difficult to relax”). The participants rated how often each emotional state applied to them over the last week on a four-point scale ranging from 0 (did not apply to me at all) to 3 (applied to me very much). The sum score of each subscale classifies the participants into one of five categories (normal, mild, moderate, severe, and extremely severe).

The CAARS and DASS questionnaires have a high reliability and validity and are suited for the dimensional approach on psychopathology [22, 23, 57].

### 2.3. Banich Letter Matching Tasks

**Figure 1:**
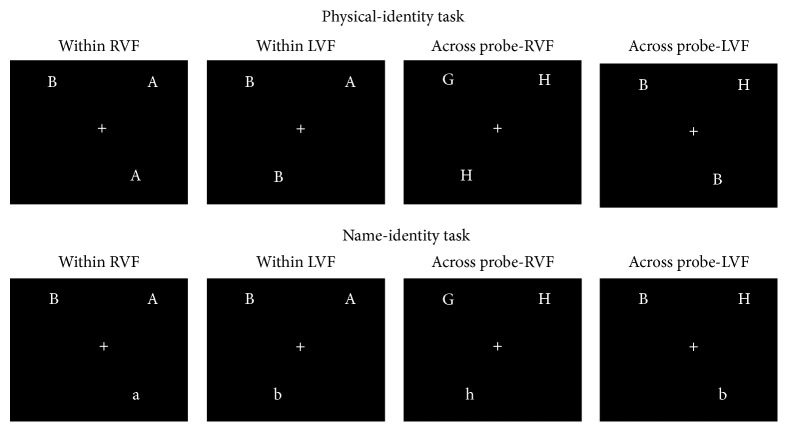
Samples of stimuli in the match trials for each task. LVF = left visual field; RVF = right visual field.

A single target letter, displayed on the left or on the right visual field below the fixation cross, had to be matched with one of two probe letters displayed laterally above the fixation cross. In the physical-identity task, all letters were displayed in upper-case and a match was defined in terms of physical-identity. In the name-identity task, the target letter was displayed in lower-case and the probe letters were displayed in upper-case, and a match was defined in terms of name-identity. Each task consisted of 80 trials; match and mismatch ratio was 50 : 50. Mismatch trials were included to prevent impulsive and careless responding. For match trials, half were within hemisphere trials in which matching letters were presented within the same visual field (10 trials per visual field of the matching probe letters). The other half of the match trials were across hemisphere trials in which matching letters were presented across visual fields (10 trials per visual field of the matching probe above the fixation cross).  Figure 1 presents stimuli in the two types of the match trials for the physical- and the name-identity task.

Each trial started with a black slide with a fixation cross presented for 1000 ms. Thereafter, three letters were presented along with the fixation cross for 150 ms. Next, a black slide with a fixation cross was assigned for participant's response for 2000 ms. Finally, a blank black slide was presented for 500 ms indicating the end of the trial.

#### 2.3.1. Stimuli

In the physical- and the name-identity task, letters were presented in upper- and lower-case from A, B, D, G, H, E, F, L, R, M, T, and Q. They were arranged in triangular position: Two different probe letters were presented 1.6° above the fixation cross, one on each visual field, 2.68° to the left or right of the midline, and the target letter was displayed 1.6° below the fixation cross, 1.6° to the left or right of the midline. The fixation cross was always located in the center of the screen during the trial. All letters had the same dimensions of 0.95° horizontally and 1.3° vertically presented in white color on a black background (to reduce the light emitted from LED screen).

#### 2.3.2. Apparatus

The tasks were conducted on a laptop computer using E-Prime software version 2.0 to control the stimulus presentation and to specify the correct responses. The letters were displayed on LED-backlit HD antiglare screen with 1024 × 768 pixel resolution and refresh rate of 60 Hz. A chin rest was used to fix the distance of 50 cm between the screen and participant's eye. A response box was used to record reaction times and correct responses. The box was positioned halfway between the chin rest and the screen to enable easy reach.

### 2.4. Procedures

Participants filled in the paper-and-pencil questionnaires and performed Banich letter matching tasks in a counterbalanced order. To perform the tasks, the participants seated in a dimly lit room, their chin rested upon the chin rest. They were instructed to press a key as fast as possible when a match appeared and not to respond during a mismatch trial. In addition, the participants were instructed to gaze at the fixation cross on the laptop screen all the time and not to move their eyes away when the stimuli appeared. For eye saccades, participants had to make the anticipated saccades after key pressing. Before each task, a practice block of trials was given until a criterion was met of seven correct responses in ten consecutive trials. After reaching this criterion, the practice block automatically terminated.

### 2.5. Data Analysis

To test whether we replicate performance findings of the Banich paradigm, a repeated measures analysis of variance was performed on mean reaction times (RTs) of the correct responses. For the physical- and the more complex name-identity task, the index of interhemispheric interaction speed was individually calculated as the difference in mean RTs between within and across hemisphere trials: [(Within RT − Across RT)/overall mean RT]. Please note that a higher value reflects faster interhemispheric interaction. According to the Banich paradigm, if the task is more complex faster interhemispheric interaction is required for an optimal performance.

To determine which ADHD and mood symptoms predict the speed of interhemispheric interaction multivariable linear regression analyses were performed. Finally, groups with high and low levels of symptomatology (first and third tertile scores) were compared on the interhemispheric interaction index using a repeated measures analysis of variance.

## 3. Results

### 3.1. Questionnaires

**Figure 2:**
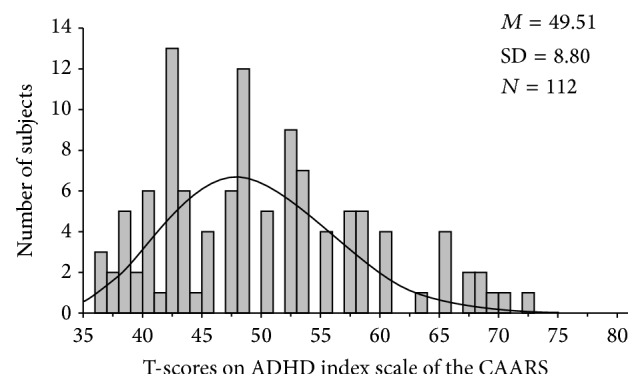
The distribution of T-scores on ADHD index subscale of the CAARS.

To save space Figure 2 shows only the distribution of the T-scores on the ADHD index as it is considered the most reliable index of ADHD. T-scores have a mean of 50 and standard deviation of 10; they are transformed from raw scores and used to compare the individual's answers to population norms [21]. The figure indicates that the index score varies along a continuum of severity and provides enough variance to test our interhemispheric interaction hypothesis using the dimensional approach. The T-score of 65 may be used as a clinical cut-off relative to the population. As can be seen, about 10% of the sample had ADHD index score of 65 (four participants) and higher (seven participants). In addition, answers on the questionnaire might be considered reliable because only 15 subjects had a score above seven on the inconsistency index of the CAARS purported to identify random or careless responding.

Like the CAARS, also the DASS is a quantitative measure with cut-off scores to characterize degree of severity relative to the population. The large majority of the sample scored within the normal range on severity (i.e., the categories normal plus mild) of mood symptoms; about 15% of the sample had high mood symptoms (see Table 2).

Pearson correlations between the overall ADHD symptomatology (ADHD index) and mood symptoms were *r* = 0.57, *p* < 0.001 for depression, *r* = 0.37, *p* < 0.001 for anxiety, and *r* = 0.50, *p* < 0.001 for stress indicating a moderate overlap of ADHD and mood symptomatologies.

### 3.2. Banich Letter Matching Tasks

Less than 5% errors were made; therefore, number of errors has not been taken into consideration. The reaction time analyses were run with and without the 15 subjects with inconsistent responding on the CAARS and run with and without the seven participants with mixed handedness. The outcomes did not essentially differ. Thus, data analyses on 112 subjects are presented below.

Pearson correlations were calculated between the overall mean RT (using the physical- and the name-identity task) and the ADHD index, inattention, hyperactivity, impulsivity, depression, anxiety, and stress. Only the ADHD index showed a positive trend with the overall mean RT (*r* = 0.17, *p* = 0.07). The finding indicates the higher the ADHD index score, the slower the RT performance. This was especially the case in the name-identity task (*r* = 0.20, *p* = 0.03) and more specifically for within hemisphere trials (*r* = 0.22, *p* = 0.01).

**Figure 3:**
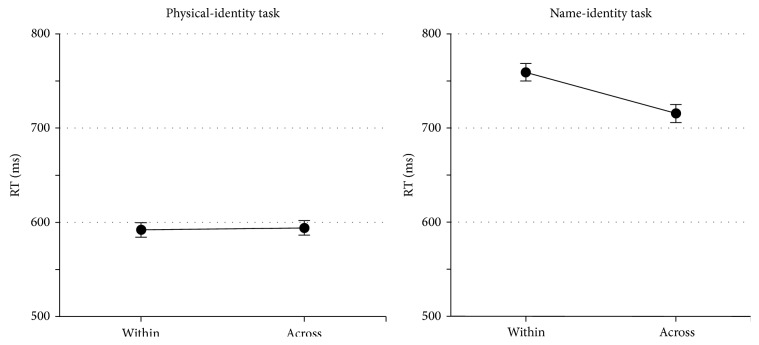
Mean reaction times (RTs) for the physical- and the name-identity task. Within = within hemisphere trials; across = across hemisphere trials; error bars indicate standard error.

Because of the imbalance gender ratio in the sample, we have tested for gender differences. Neither the main effect of gender, on reaction time performance, nor its interactions with task nor trial type were significant (*p* ≥ 0.27). Therefore, we collapsed the mean RTs for males and females together (see Figure 3).

To investigate whether our reaction time data could be interpreted in terms of the Banich paradigm, a repeated measures analysis of variance was carried out on reaction time performance. The within subjects factors were* task* (physical-identity, name-identity) and* trial type* (within hemisphere, across hemisphere). The analysis revealed a significant main effect of task,* F* (1, 111) = 421.01, *p* < 0.001, *η* ^2^_*p*_ = 0.79, with faster RTs in the physical-identity task (*M* = 593) than the name-identity task (*M* = 743). According to the Banich paradigm, the interaction between trial type and task must be significant. This was the case in our data set: the interaction was* F* (1, 111) = 40.96, *p* < 0.001, *η* ^2^_*p*_ = 0.27; the RTs in the name-identity task were faster on across hemisphere trials (*M* = 721) compared to within hemisphere trials (*M* = 766), while RTs in the physical-identity task were equal for within hemisphere (*M* = 592) and across hemisphere trials (*M* = 593).

**Figure 4:**
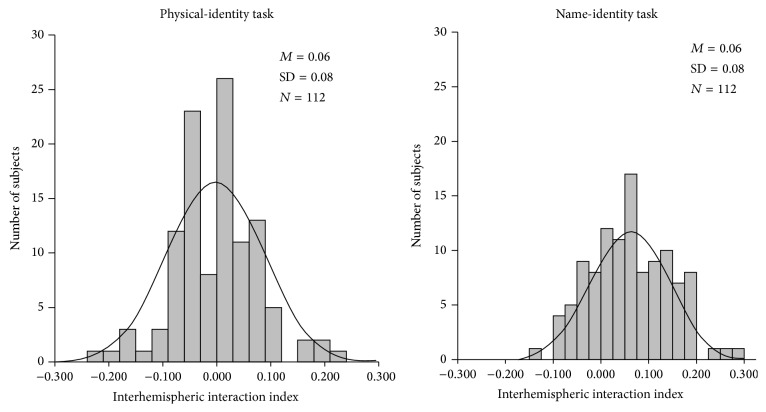
The distribution of interhemispheric interaction index scores for the physical- and the name-identity task. Interhemispheric interaction index = [(mean RT of within hemisphere trials − mean RT of across hemisphere trials)/overall mean RT].

The left and right panels of Figure 4 present the distribution of interhemispheric interaction speed index (difference in mean RTs between within and across hemisphere trials) of, respectively, the physical- and the name-identity task. Both panels together show that the indices were normally distributed, statistically confirmed by the nonsignificant Shapiro-Wilk tests (*W* = 0.98, *p* = 0.08 for the physical-identity task and *W* = 0.99, *p* = 0.71 for the name-identity task).

The interhemispheric interaction speed indices for both tasks were used as dependent variables in two multivariable linear regression analyses. In the first, the ADHD index was the independent variable. The results indicated that the ADHD index was not a predictor (*R* ^2^ ≤ 0.03, *p* ≥ 0.36). In the second, the three key domains of ADHD (inattention, hyperactivity, and impulsivity) were the independent variables. The results revealed nonsignificant predictors (*R* ^2^ ≤ 0.04, *p* ≥ 0.28). Consequently, the ADHD symptomatology in isolation from mood symptomatology has no association with interhemispheric interaction. The picture becomes different when exploring the combination of both symptomatologies. Tables 3 and 4 present regression analyses including the aforementioned ADHD subscales with a backward selection of the DASS mood subscales. The resulting models were significant only in the name-identity task. The key domains of ADHD symptoms and stress explained about 10% of the variance of interhemispheric interaction index. Here the ADHD symptoms (especially hyperactivity) and stress were significant predictors of, respectively, fast and slow interhemispheric interaction.

In sum, on the basis of regression analyses it is concluded that only the combination of ADHD symptoms and stress is linked with the speed of interhemispheric interaction.

To explore in detail the effect of stress on ADHD symptoms and the speed of interhemispheric interaction, Pearson correlations were calculated between the ADHD index, hyperactivity scores, and the interhemispheric interaction index of the name-identity task in the group with high stress (score >14 on the stress subscale; *n* = 33) and in the group with low stress (score ≤14; *n* = 79) apart. In the group with high stress, faster interhemispheric interaction was correlated with higher scores on the ADHD index (*r* = 0.37, *p* = 0.03) and the hyperactivity subscale (*r* = 0.41, *p* = 0.02). In the group with low stress correlations were not significant with *p* values ≥0.39.

Finally, to test the ADHD-interhemispheric interaction link more thoroughly we compared the speed of the interhemispheric interaction of first and third tertile groups on the ADHD index, inattention, hyperactivity, and impulsivity subscales of the CAARS. Repeated measures analyses of variance with a within subject factor of* task* (physical-identity, name-identity) and a between subjects factor of* group* (low-score, high-score) indicated that group composition based on the ADHD index, inattention, and impulsivity subscales revealed no group differences. However, the high-score group on the hyperactivity subscale showed overall faster interhemispheric interaction than the low-score group; the main effect of group was significant* F* (1, 74) = 3.95, *p* < 0.05, *η* ^2^_*p*_ = 0.06. Post hoc analysis for the hyperactivity subscale indicated that the groups differed in the name-identity task,* t* (74) = −2.17, *p* < 0.03, but not in the physical-identity task; the mean interhemispheric interaction indices were 0.046 and 0.086 in the name-identity task, and they were −0.006 and 0.010 in the physical-identity task for, respectively, the low- and the high-score group.

## 4. Discussion

The present study is, to the best of our knowledge, the first to use the dimensional approach to investigate the relation between interhemispheric interaction and ADHD symptomatology. In many ways, the participating sample is more homogenous compared to samples of clinical studies on this subject with respect to factors such as age and IQ. Known mood comorbidities have been controlled for. Other potentially relevant comorbidities related to ADHD such as learning and conduct disorders are supposed to be absent in the participating university student sample.

The task used may be considered a valid and reliable instrument to estimate interhemispheric interaction. First, as expected from the Banich paradigm, the more complex name-identity task needed more information processing resources resulting in slower reaction times compared to the less complex physical-identity task. Second, across hemisphere advantage was found in the name-identity task indicating that the performance benefits from interhemispheric interaction when tasks are more complex. Consequently, the two performance findings were in accordance with literature.

The main conclusion was that ADHD symptomatology, especially hyperactivity, together with stress contributes weakly but significantly to the speed of interhemispheric interaction, at least as far as visual information processing is concerned suggesting that callosal splenium (the main structural pathways that transfer visual information between the two hemispheres [58]) function might be affected. For ADHD symptomatology-only no relationship was found.

It might be surprising that only hyperactivity was playing a role in our study, not the other symptoms, because longitudinal studies have indicated that hyperactivity decreases whereas inattention, impulsivity increase as a function of age [59]. One possibility is that, compared to other samples, university students even those with high hyperactivity scores are not likely to report poor attention/impulsivity. An alternative possibility is that the three domains of ADHD symptoms are related to different neurobiological variables [10, 60, 61] and that hyperactivity is exclusively related to fast interhemispheric interaction. For example, Buchmann et al. [62] suggested that motor hyperactivity could be induced by disturbances of myelination of transcallosal fibres in children with ADHD and possibly the disturbances may remain into adulthood in one-third of the cases.

The fact that stress plays a crucial role in the dimensional relationship between ADHD symptoms and interhemispheric communication gives further support to the idea that ADHD is a stress-related disorder which might be one of the important causes of ADHD [48]. Please note that Wender [63] already proposed to add stress as an additional criterion of the adulthood ADHD diagnosis.

The present findings indicate that ADHD symptomatology is related to slower mean RT and that the ADHD symptomatology in the presence of stress is linked to faster interhemispheric interaction. At first sight, faster interhemispheric interactions could be interpreted in positive terms. This would be the case if fast interhemispheric interaction is linked to fast performance. However, in our sample RT performance of subjects with higher level of ADHD symptoms did not benefit from fast interhemispheric interaction: they were overall slower. Our findings suggest that optimal performance requires a specific range of interhemispheric interaction speed and that too fast or too slow interhemispheric interaction may negatively affect or interfere with performance. Within the Banich paradigm, the optimal range of interhemispheric interaction may vary as a function of the level of task complexity. Consequently, our findings justify suggesting that ADHD symptomatology is linked with fast but nonoptimal interhemispheric interaction. The finding that ADHD symptomatology is related to faster interhemispheric interaction is in line with clinical studies [8–10], but unfortunately, studies did not report the extent of overlap between ADHD and stress.

## 5. Limitations and Future Research

The first limitation of the present study could have been the lack of eye-gaze verification. Central fixation was encouraged and participants were instructed to make eye saccades after key pressing. Nevertheless, it is possible that participants' gaze was not directed to the central fixation cross on every trial. We have to admit that these variations in gaze could have influenced performance. However, given the low error percentage together with fast stimulus presentation (150 ms), it seems unlikely that variations in gaze confounded our results.

A second limitation is that the university students are not representative with the adult population in general. In addition, the present sample includes more females than males. As a result, the present outcome needs replication for adults in general and should also examine samples with more equal sex distribution. Having this said, the CAARS scores are corrected for gender and the reaction time data showed no difference between male and female students. It is well-recognized that the validity of self-reports from students may be questioned. For instance, one should be suspicious if students rate themselves as being significantly symptomatic yet have managed to achieve well in school and in other life activities, especially, if their T-scores were above 80 [64]. Please note that in our sample the majority responded in a valid and reliable way with scores within the population mean.

A third limitation is that our data apply only to right-handers. The relation between ADHD symptoms and interhemispheric interaction remains unexplored in inconsistent- and left-handers; this calls for future studies on inconsistent-handers specifically as it has been reported that inconsistent handedness is associated with increased interhemispheric interaction [65] and ADHD [16].

The present study focused on integrating visual information processing (subserved by the posterior part of the corpus callosum). It is recommended to direct future research towards the anterior part of the corpus callosum connecting the frontal and prefrontal cortex (i.e., genu). This structure subserves higher order cognition such as executive function and its possible interactions with self-regulation and effort which are assumed to be compromised in ADHD [66–68].

## 6. Conclusions

Our dimensional findings indicated that ADHD symptomology, especially hyperactivity, in the presence of stress was weakly but significantly associated with fast interhemispheric interaction. These findings are supporting studies on clinical samples.

## Figures and Tables

**Table 1:** Characteristics of study sample.

Gender	30 males, 82 females

Age	M = 20.3, SD = 2.3 (min : max = 18 : 32 years)

Handedness	M = 69.41, SD = 22.72 (min : max = 12 : 100)

Participants with DSM diagnoses	Eight participants have reported a diagnosis (two with anxiety and depression; three with ADHD; one with both ADHD and depression; and two with ADD and depression)

**Table 2:** Number of subjects scoring in various ranges on the DASS subscales.

DASS subscales	Normal	Mild	Moderate	Severe	Extremely severe
Depression	93	10	3	5	1
Anxiety	81	11	15	4	1
Stress	79	11	19	3	0

**Table 3:** The final models of multivariable regression analyses predicting interhemispheric interaction index in each task from the ADHD index subscale of the CAARS and the backward selection of the DASS subscales.

Task	Predictor	*β*	*R* ^2^	Adjusted *R* ^2^
Physical-identity	ADHD index	−0.052	0.003	−0.006

Name-identity	ADHD index	0.201^†^	0.068^∗^	0.051^∗^
	Stress	−0.295^∗^		

*Note*. ^†^ *p* < 0.07, ^∗^ *p* < 0.01.

**Table 4:** The final models of multivariable regression analyses predicting interhemispheric interaction index in each task from inattention, hyperactivity, and impulsivity subscales of the CAARS and the backward selection of the DASS subscales.

Task	Predictor	*β*	*R* ^2^	Adjusted *R* ^2^
Physical-identity	Inattention	−0.029	0.029	0.002
	Hyperactivity	0.188		
	Impulsivity	−0.166		

Name-identity	Inattention	−0.046	0.102^∗^	0.068^∗^
	Hyperactivity	0.226^∗^		
	Impulsivity	0.230		
	Stress	−0.332^∗^		

*Note*. ^∗^ *p* < 0.01.

## References

[1] Kofler M. J., Rapport M. D., Sarver D. E. (2013). Reaction time variability in ADHD: a meta-analytic review of 319 studies. *Clinical Psychology Review*.

[2] Hart H., Radua J., Mataix-Cols D., Rubia K. (2012). Meta-analysis of fMRI studies of timing in attention-deficit hyperactivity disorder (ADHD). *Neuroscience & Biobehavioral Reviews*.

[3] Jacobs R., Harvey A. S., Anderson V. (2011). Are executive skills primarily mediated by the prefrontal cortex in childhood? Examination of focal brain lesions in childhood. *Cortex*.

[4] Schulte T., Müller-Oehring E. M. (2010). Contribution of callosal connections to the interhemispheric integration of visuomotor and cognitive processes. *Neuropsychology Review*.

[5] Woodward L. J., Clark C. A. C., Pritchard V. E., Anderson P. J., Inder T. E. (2011). Neonatal white matter abnormalities predict global executive function impairment in children born very preterm. *Developmental Neuropsychology*.

[6] Hutchinson A. D., Mathias J. L., Banich M. T. (2008). Corpus callosum morphology in children and adolescents with attention deficit hyperactivity disorder: a meta-analytic review. *Neuropsychology*.

[7] van Linschoten R., Clydesdale D., Dierckx B., Mous S. E. (2013). Midline surface area of the corpus callosum in children with attention-deficit/hyperactivity disorder: a systematic review. *Erasmus Journal of Medicine*.

[8] Brown L. N., Vickers J. N. (2004). Temporal judgments, hemispheric equivalence, and interhemispheric transfer in adolescents with attention deficit hyperactivity disorder. *Experimental Brain Research*.

[9] Hagelthorn K. M. (1998). *Attention-deficit hyperactivity disorder and functioning of the corpus callosum as measured by event-related potentials [Ph.D. thesis]*.

[10] Rolfe M. H. S., Kirk I. J., Waldie K. E. (2007). Interhemispheric callosal transfer in adults with attention-deficit/hyperactivity disorder: an event-related potential study. *NeuroReport*.

[11] Amano S. S. (2000). *Callosal functioning in children with attention deficit hyperactivity disorder [Ph.D. thesis]*.

[12] Hale T. S., McCracken J. T., McGough J. J., Smalley S. L., Phillips J. M., Zaidel E. (2005). Impaired linguistic processing and atypical brain laterality in adults with ADHD. *Clinical Neuroscience Research*.

[13] Hale T. S., Smalley S. L., Hanada G. (2009). Atypical alpha asymmetry in adults with ADHD. *Neuropsychologia*.

[14] Hale T. S., Zaidel E., McGough J. J., Phillips J. M., McCracken J. T. (2006). Atypical brain laterality in adults with ADHD during dichotic listening for emotional intonation and words. *Neuropsychologia*.

[15] Hudziak J. J., Achenbach T. M., Althoff R. R., Pine D. S. (2007). A dimensional approach to development psychopathology. *International Journal of Methods in Psychiatric Research*.

[16] Rodriguez A., Kaakinen M., Moilanen I. (2010). Mixed-handedness is linked to mental health problems in children and adolescents. *Pediatrics*.

[17] Parens E., Johnston J. (2009). Facts, values, and attention-deficit hyperactivity disorder (ADHD): an update on the controversies. *Child and Adolescent Psychiatry and Mental Health*.

[18] Verdoux H., van Os J. (2002). Psychotic symptoms in non-clinical populations and the continuum of psychosis. *Schizophrenia Research*.

[19] Barkley R. A., Barkley R. A. (2006). Primary symptoms, diagnostic criteria, prevalence and gender differences. *Attention-Deficit Hyperactivity Disorder: A Handbook for Diagnosis and Treatment*.

[20] Marcus D. K., Barry T. D. (2011). Does attention-deficit/hyperactivity disorder have a dimensional latent structure? A taxometric analysis. *Journal of Abnormal Psychology*.

[21] Conners C. K., Erhardt D., Sparrow E. (1999). *Conners' Adult ADHD Rating Scales (CAARS) Technical Manual*.

[22] Adler L. A., Faraone S. V., Spencer T. J. (2008). The reliability and validity of self- and investigator ratings of ADHD in adults. *Journal of Attention Disorders*.

[23] Erhardt D., Epstein J. N., Conners C. K., Parker J. D. A., Sitarenios G. (1999). Self-ratings of ADHD symptomas in auts II: reliability, validity, and diagnostic sensitivity. *Journal of Attention Disorders*.

[24] Kooij J. J. S., Boonstra A. M., Swinkels S. H. N., Bekker E. M., de Noord I., Buitelaar J. K. (2008). Reliability, validity, and utility of instruments for self-report and informant report concerning symptoms of ADHD in adult patients. *Journal of Attention Disorders*.

[25] Magnússon P., Smári J., Sigurdardóttir D. (2006). Validity of self-report and informant rating scales of adult ADHD symptoms in comparison with a semistructured diagnostic interview. *Journal of Attention Disorders*.

[26] Malkovsky E., Merrifield C., Goldberg Y., Danckert J. (2012). Exploring the relationship between boredom and sustained attention. *Experimental Brain Research*.

[27] Alexander S. J., Harrison A. G. (2013). Cognitive responses to stress, depression, and anxiety, and their relationship to ADHD symptoms in first year psychology students. *Journal of Attention Disorders*.

[28] Steer R. A., Ranieri W. F., Kumar G., Beck A. T. (2003). Beck Depression Inventory-II items associated with self-reported symptoms of ADHD in adult psychiatric outpatients. *Journal of Personality Assessment*.

[29] Herrmann M. J., Saathoff C., Schreppel T. J. (2009). The effect of ADHD symptoms on performance monitoring in a non-clinical population. *Psychiatry Research*.

[30] Hoogman M., Rijpkema M., Janss L. (2012). Current self-reported symptoms of attention deficit/hyperactivity disorder are associated with total brain volume in healthy adults. *PLoS ONE*.

[31] Banich M. T., Belger A. (1990). Interhemispheric interaction: how do the hemispheres divide and conquer a task?. *Cortex*.

[32] Banich M. T., Passarotti A. M., Janes D. (2000). Interhemispheric interaction during childhood: I. Neurologically intact children. *Developmental Neuropsychology*.

[33] Banich M. T., Passarotti A. M., White D. A., Nortz M. J., Steiner R. D. (2000). Interhemispheric interaction during childhood: II. Children with early-treated phenylketonuria. *Developmental Neuropsychology*.

[34] Bourne V. J. (2006). The divided visual field paradigm: methodological considerations. *Laterality*.

[35] Marzi C. A. (2010). Asymmetry of interhemispheric communication. *Wiley Interdisciplinary Reviews: Cognitive Science*.

[36] Pollmann S., Zaidel E., von Cramon D. Y. (2003). The neural basis of the bilateral distribution advantage. *Experimental Brain Research*.

[37] Banich M. T., Brown W. S. (2000). A life-span perspective on interaction between the cerebral hemispheres. *Developmental Neuropsychology*.

[38] Bayer U., Kessler N., Güntürkün O., Hausmann M. (2008). Interhemispheric interaction during the menstrual cycle. *Neuropsychologia*.

[39] Lopez M., Kosson D. S., Weissman D. H., Banich M. T. (2007). Interhemispheric integration in psychopathic offenders. *Neuropsychology*.

[40] Markee T., Brown W. S., Moore L. H., Theberge D. C. (1996). Callosal function in dyslexia: evoked potential interhemispheric transfer time and bilateral field advantage. *Developmental Neuropsychology*.

[41] Welcome S. E., Chiarello C. (2008). How dynamic is interhemispheric interaction? Effects of task switching on the across-hemisphere advantage. *Brain and Cognition*.

[42] Belger A., Banich M. T. (1992). Interhemispheric interaction affected by computational complexity. *Neuropsychologia*.

[43] Belger A., Banich M. T. (1998). Costs and benefits of integrating information between the cerebral hemispheres: a computational perspective. *Neuropsychology*.

[44] Weissman D. H., Banich M. T. (2000). The cerebral hemispheres cooperate to perform complex but not simple tasks. *Neuropsychology*.

[45] Combs M. A., Canu W. H., Broman-Fulks J. J., Rocheleau C. A., Nieman D. C. (2012). Perceived stress and ADHD symptoms in adults. *Journal of Attention Disorders*.

[46] Howard D., Schiraldi G., Pineda A., Campanella R., Landow M. V. (2006). Stress and mental health among college students: overview and promising prevention interventions. *Stress and Mental Health of College Students*.

[47] Whalen C. K., Jamner L. D., Henker B., Delfino R. J., Lozano J. M. (2002). The ADHD spectrum and everyday life: experience sampling of adolescent moods, activities, smoking, and drinking. *Child Development*.

[48] Grosswald S. J., Banerjee S. (2013). Is ADHD a stress-related disorder? Why meditation can help. *Attention Deficit Hyperactivity Disorder in children and Adolescents*.

[49] Compton R. J., Mintzer D. A. (2001). Effects of worry and evaluation stress on interhemispheric interaction. *Neuropsychology*.

[50] Bajwa S., Bermpohl F., Rigonatti S. P., Pascual-Leone A., Boggio P. S., Fregni F. (2008). Impaired interhemispheric interactions in patients with major depression. *The Journal of Nervous and Mental Disease*.

[51] Compton R. J., Wilson K., Wolf K. (2003). When two hemispheres are not better than one: effects of worry on interhemispheric processing. *Brain and Cognition*.

[52] Compton R. J., Wilson K., Wolf K. (2004). Mind the gap: interhemispheric communication about emotional faces. *Emotion*.

[53] Han K.-M., Choi S., Jung J. (2014). Cortical thickness, cortical and subcortical volume, and white matter integrity in patients with their first episode of major depression. *Journal of Affective Disorders*.

[54] Lovibond P. F., Lovibond S. H. (1995). The structure of negative emotional states: Comparison of the depression anxiety stress scales (DASS) with the Beck Depression and Anxiety Inventories. *Behaviour Research and Therapy*.

[55] Oldfield R. C. (1971). The assessment and analysis of handedness: the Edinburgh inventory. *Neuropsychologia*.

[56] López-Arvizu C., Sparrow E. P., Strube M. J. (2011). Increased symptoms of attention deficit hyperactivity disorder and major depressive disorder symptoms in nail-patella syndrome: potential association with LMX1B loss-of-function. *American Journal of Medical Genetics, Part B: Neuropsychiatric Genetics*.

[57] Antony M. M., Cox B. J., Enns M. W., Bieling P. J., Swinson R. P. (1998). Psychometric properties of the 42-item and 21-item versions of the Depression Anxiety Stress Scales in clinical groups and a community sample. *Psychological Assessment*.

[58] Knyazeva M. G. (2013). Splenium of corpus callosum: patterns of interhemispheric interaction in children and adults. *Neural Plasticity*.

[59] Wilens T. E., Biederman J., Spencer T. J. (2002). Attention deficit/hyperactivity disorder across the lifespan. *Annual Review of Medicine*.

[60] Diamond A. (2005). Attention-deficit disorder (attention-deficit/hyperactivity disorder without hyperactivity): a neurobiologically and behaviorally distinct disorder from attention-deficit/hyperactivity disorder (with hyperactivity). *Development and Psychopathology*.

[61] Milich R., Balentine A. C., Lynam D. R. (2001). ADHD combined type and ADHD predominantly inattentive type are distinct and unrelated disorders. *Clinical Psychology: Science and Practice*.

[62] Buchmann J., Wolters A., Haessler F., Bohne S., Nordbeck R., Kunesch E. (2003). Disturbed transcallosally mediated motor inhibition in children with attention deficit hyperactivity disorder (ADHD). *Clinical Neurophysiology*.

[63] Wender P. H. (1995). *Attention-Deficit-Hyperactivity-Disorder in Adults*.

[64] Harrison A. G., Edwards M. J., Parker K. C. H. (2007). Identifying students faking ADHD: preliminary findings and strategies for detection. *Archives of Clinical Neuropsychology*.

[65] Prichard E., Propper R. E., Christman S. D. (2013). Degree of handedness, but not direction, is a systematic predictor of cognitive performance. *Frontiers in Psychology*.

[66] Sergeant J. A. (2005). Modeling attention-deficit/hyperactivity disorder: a critical appraisal of the cognitive-energetic model. *Biological Psychiatry*.

[67] Sonuga-Barke E. J., van der Meere J. J. W., Roeyers H. (2010). Context-dependent dynamic processes in attention deficit/hyperactivity disorder: differentiating common and unique effects of state regulation deficits and delay aversion. *Neuropsychology Review*.

[68] Van der Meere J. J., Borger N. A., Wiersema R. J. (2010). ADHD: state regulation and motivation. *Current Medical Literature—Psychiatry*.

